# Influence of the Treatment Temperature on the Microstructure and Hydration Behavior of Thermoactivated Recycled Cement

**DOI:** 10.3390/ma13183937

**Published:** 2020-09-05

**Authors:** Sofia Real, Ana Carriço, José Alexandre Bogas, Mafalda Guedes

**Affiliations:** 1 Civil Engineering Research and Innovation for Sustainability, Instituto Superior Técnico, Universidade de Lisboa, Av. Rovisco Pais, 1049-001 Lisbon, Portugal; ana.carrico@tecnico.ulisboa.pt (A.C.); jose.bogas@tecnico.ulisboa.pt (J.A.B.); 2 Center for Product Development and Technology Transfer and Department of Mechanical Engineering, Setúbal School of Technology, Instituto Politécnico de Setúbal, 2910-761 Setúbal, Portugal; mafalda.guedes@estsetubal.ips.pt; 3 Center of Physics and Engineering of Advanced Materials, Instituto Superior Técnico, Av. Rovisco Pais, 1049-001 Lisboa, Portugal

**Keywords:** recycled cement, cement thermal activation, cement rehydration

## Abstract

This paper intends to contribute to a better knowledge of the production and rehydration of thermoactivated recycled cement and its incorporation in cement-based materials. To this end, the influence of the treatment temperature on the properties of recycled cements and recycled cement pastes was assessed by means of a wide array of tests. Anhydrous recycled cement as well as the resulting pastes were characterized through density and particle size, water demand and setting time, thermogravimetry, X-ray diffraction, field emission gun scanning electron microscopy, isothermal calorimetry, ^29^Si nuclear magnetic resonance spectroscopy, flowability, mechanical strength, mercury intrusion porosimetry and scanning electron microscopy. The treatment temperature had a significant influence on the dehydration and hydration of recycled cement, essentially resulting in the formation of C_2_S polymorphs of varying reactivity, which led to pastes of different fresh and hardened behaviors. The high water demand and the pre-hydration of recycled cement resulted in high setting times and low compressive strengths. The highest mechanical strength was obtained for a treatment temperature of 650 °C.

## 1. Introduction

Over the past decades, concern regarding the sustainable development of the construction industry has led to the search for new more eco-efficient solutions, addressing some of the main environmental issues introduced by concrete production [1]. These include natural resources depletion, energy consumption, greenhouse gas emissions, as well as the worldwide production of high volume of construction and demolition waste [1,2]. The significant economic and environmental impact therefore legitimates concrete recycling and materials circular economy as a priority in the construction industry.

Though concrete recycling has long been investigated, the focus has essentially remained on its direct use in the replacement of natural aggregates or as a low-activity filler addition in partial cement replacement [3]. Recently, efforts have been made towards the recovery of the cementitious part of waste concrete through thermal activation. in order to explore the rehydration ability of thermoactivated recycled cement (RC) [1,4]. Some authors simply concentrated on a single treatment temperature [5,6] and others approached only a few properties, namely the fresh behavior and the mechanical strength [4,7,8,9,10,11,12,13]. Other studies were dedicated to the incorporation of varying percentages of RC in mortars and concrete [14,15,16,17,18,19,20,21,22]. Furthermore, results reported in the literature are contradictory, reflecting the lack of knowledge of these poorly explored new binders [1].

Two particularities of RC seem to be the high water demand [4,5,6,8,23,24] and reduced setting time [4,8]. However, the cause as well as the influence of the treatment temperature on these properties has yet to gather consensus. Shui et al. [4] and Xuan and Shui [13] found a linear increase of the water demand with the treatment temperature, between 300 and 900 °C, reporting pastes with water-to-binder ratios (w/b) ranging about 0.48–0.68. A similar tendency was reported by Vysvaril et al. [8], though higher w/b were obtained for the same array of temperatures (about 0.7–0.9 for 200–800 °C). Baldusco et al. [5] ascribed the results to the high surface area of RC. Shui et al. [4] and Xuan and Shui [13] mentioned that, besides the high surface area, the high water demand was also owed to the free CaO content of RC. Xinwei et al. [23], and Zhang et al. [6] attributed this to RC particle absorption, whereas Yu and Shui [24] also highlighted the effect of particle agglomeration.

The rapid setting times of RC have been attributed to their fast rehydration, owed to their reactivity and surface area [4,5,6]. Moreover, the decrease of the setting time with the treatment temperature has been described by various authors [4,5,6,8,13], for treatment temperatures up to about 800 °C, though different results were reported. Diverging reasons for this have been presented, namely the increasing dehydration of RC [4] or growing CaO content [8,14]. However, Serpell and Lopez [14] attributed this apparent rapid setting to a false set due to the quick hydration of CaO. Moreover, higher setting times in RC than in ordinary Portland cement (PC) were found by Bogas et al. [15] and Carriço et al. [22]. This less expected behavior was attributed to the agglomeration of RC particles and the higher pre-hydration propensity of RC, during cooling and storage.

The phase composition of the dehydrated cement with thermal treatment still has not been fully comprehended. Most studies have only been able to identify that after thermal treatment, C_2_S of varying reactivity are generated [5,8,9,10]. Through nuclear magnetic resonance spectroscopy (^29^Si-NMR), Alonso and Fernandez [10] determined that the thermal treatment of the cement above 200 °C resulted in a new nesosilicate, which was less crystalline than the C_2_S of the original cement. Combining X-ray diffraction analysis (XRD) with ^29^Si-NMR, Lü et al. [12] inferred that, above 650 °C, β-C_2_S was formed, and at 900 °C, this polymorph became highly crystalline and had low reactivity. Serpell and Zunino [11] reported that β-C_2_S and a more reactive C_2_S polymorph, α’_H_-C_2_S, were identified above 740 °C, though, up to 800 °C, the predominant C_2_S polymorph was α’_H_-C_2_S, and, at 900 °C, the main C_2_S polymorph was β-C_2_S. Shui et al. [25] identified α-C_2_S, as well as β-C_2_S, above 800 °C. Through XRD analysis, Carriço et al. [22] detected that, above 600 °C, a new C_2_S polymorph was created, with a similar structure to α’_L_-C_2_S. Furthermore, the microstructure and hydration mechanism of RC has yet to be completely figured out, especially taking into account the influence of the treatment temperature. The initial hydration heat of RC was found to be significantly higher than that of ordinary PC [4,5,6,26], though the reasons for this phenomenon are yet to be entirely understood. Shui et al. [4] associated this phenomenon to the high surface area and dehydrated compound instability of RC. Baldusco et al. [5] added that the high calcium aluminate content of RC also contributed to this. Other authors connected the results to the exothermic reaction of CaO hydration [9,15,26]. Regarding the microstructure development, only some qualitative analysis, essentially based on scanning electron microscopy analysis (SEM), have been carried out for a limited range of treatment temperatures [4,5]. A more detailed review of the current state-of-the-art in recycled cements is presented in Carriço et al. [1].

In sum, although thermoactivated recycled cement has emerged as a prospective eco-efficient alternative to current binders, the knowledge on this domain remains scarce and key aspects related to its production, hydration, microstructure, fresh and hardened behavior are still not fully understood.

This paper intends to contribute to a better knowledge of the production, rehydration and microstructure of thermoactivated recycled cement, affected by the thermal treatment temperature. Based on an extensive experimental campaign, cement pastes produced with waste cement treated at different temperatures (400–900 °C) were characterized in terms of their rehydration behavior, microstructure and mechanical properties. For this purpose, thermogravimetry (TG), X-ray diffraction (XRD), field emission gun scanning electron microscopy (FEG-SEM), nuclear magnetic resonance spectroscopy (^29^Si-NMR), isothermal calorimetry (IC), mercury intrusion porosimetry (MIP) and scanning electron microscopy (SEM), as well as water demand, setting time and mechanical strength tests were performed on RC pastes and also on PC pastes (for comparison purposes).

## 2. Experimental Program

### 2.1. Production of Recycled Cements

A reference waste cement was first prepared, to be used as starting material throughout all further experiments. For that purpose, a CEM I 42.5R cement (SECIL, Setúbal, Portugal) (Table 1) was used to produce an origin paste (OP) with a w/b of 0.55, which displayed an average compressive strength at 28 days of approximately 41 MPa. The OP was cured in water until 7 days, after which it was kept in laboratory environment conditions at an average relative humidity (RH) of 60–70%. After at least three months, the OP was comminuted through a sequence of crushing, grinding and milling operations. The obtained waste cement was first reduced down to particles of about 7 cm by means of two jaw crushers with different jaw openings, followed by two roll milling steps, leading to a particle size below 2 mm. Afterwards, the particles were further ground in a horizontal steel ball mill for about 2 h and sieved with a 250 μm cut-off. The obtained fines were labelled NT (non-thermoactivated waste cement) and used in all further experiments.

The thermal activation of the waste cement was carried out in a rotary tube furnace from Thermolab Scientific Equipments, by heating at 10 °C/min up to the treatment temperature. The tested temperatures were 400, 450, 500, 600, 650, 700, 750, 800 and 900 °C, covering a range from the beginning of calcium hydroxide (CH) phase dehydration to the end of cement decarbonation (at which point the eco-efficiency and rehydration activity of RC is expected to decrease [1,11,12]). The residence time at the treatment temperature was always 3 h, followed by cooling inside the oven until room temperature was reached. The produced materials were labelled RC400 to RC900, referring to the thermal activation temperature of the corresponding RC.

### 2.2. Formulation and Production of Recycled Cement Pastes

The obtained RC powders were used to produce recycled cement pastes, labelled P400–P900 (Table 2), where 400–900 refers to the thermal activation temperature of the recycled cement used to produce the corresponding paste (P). For each paste, a w/b was chosen to ensure normal consistency, according to EN 196-3 [30]. For comparison purposes, two reference pastes were also produced using CEM I 42.5R instead of RC, one with a w/b ensuring normal consistency (PC1) and another with the same w/b as P700 (PC2). Furthermore, an additional paste was produced with NT (PNT), in order to assess the influence of the thermal treatment on the reactivity of RC. Table 2 presents the composition of the produced pastes. All pastes were produced by mixing cement (either PC, NT or RC) with water for about 8 min, to ensure the homogenization of the mixture, effective water absorption and hydration of free CaO present in RC.

### 2.3. Characterization Methods

#### 2.3.1. Characterization of Recycled Cements

The recycled cement powders were characterized regarding their morphology, composition and rehydration capacity.

The morphological features were analyzed by field emission gun scanning electron microscopy (FEG-SEM) (JSM-7001F, Jeol, Tokyo, Japan). The samples for FEG-SEM were previously covered with a gold-palladium alloy to avoid electric charge accumulation during observation. The absolute density of the produced powders was measured by helium pycnometry (Multi, Quantachrome Instruments, Boynton Beach, FL, USA). The particle size distribution was measured by laser diffraction (Mastersizer 2000, Malvern Instruments, Malvern, UK).

The crystalline phases present before and after the thermal treatment were assessed by X-ray diffraction (XRD) of previously ground samples. A PANalytical X’Pert Pro diffractometer (Malvern Panalytical, Malvern, UK) with CuK_α_ radiation was used. The samples were scanned in the 5–60° 2θ range, with a step size of 0.03° and a step time of 100 s. The information on the samples’ composition was complemented by nuclear magnetic resonance spectroscopy (NMR), recorded at the ^29^Si resonance of 59.595 MHz, resorting to a TecMag/Bruker 300 (Bruker, Billerica, MA, USA) “wide bore” spectrometer with a spinning of approximately 5 kHz and a relaxation delay of 20 s. The number of scans was 250 and tetrakis (trimethyl sylil) silane (^29^Si = −9.8; −135.64 ppm) was used as external reference.

The thermogravimetry (TG) thermal analysis was carried out (PC LUXX Thermobalance, Netzsh, Selb, Germany) by heating each sample (approx. 40 mg) at 25 °C/min up to 1000 °C, under nitrogen gas flow (150 mL/min).

#### 2.3.2. Characterization of Recycled Cement Pastes

The RC were characterized in terms of water demand for the production of pastes with normal consistency, and corresponding flowability, setting time and hydration heat. The flowability was measured according to EN 1015-3 [31] and the water demand and setting time in pastes with normal consistency were determined according to EN 196-3 [30]. The rehydration process was monitored by IC (TAM Air—8 channels, TA Instruments, New Castle, DE, USA) at 20 °C, according to EN 196-11: Method A (external mixing) specification [32]. Samples with approx. 6 g were prepared by mixing dedicated amounts of each RC with distilled water to achieve pastes with a w/b of about 1.0, which were placed in the calorimeter less than 4 min after mixing. Each sample was tested against a reference ampoule containing the same amount of distilled water as the pastes. The released heat measurement only started after the thermal stabilization of the apparatus following sample introduction (approx. 45 min) and proceeded for 7 days.

The pastes produced with recycled cement powders were also characterized regarding their hardened properties. For each composition and age, three 160 mm × 40 mm × 40 mm specimens were produced and kept in a wet chamber at about 20 ± 2 °C and over 95% RH. The mechanical characterization of the specimens was carried out at 1, 3, 7, 28 and 90 days of age by flexural and compressive strength tests, according to EN 1015-11 [33].

After the flexural strength test, sections of about 40 mm × 40 mm × 4 mm were sliced from the intact part of the specimens and used for morphological, microstructural and compositional analyses of the hardened pastes. In this case, the hydration was stopped at the required age using the solvent exchange method by immersion in isopropanol (with periodical solvent exchange) to remove free water. The hardened samples were then oven-dried at 60 °C for 48 h and kept in a desiccator until studied. The fracture surfaces were observed by SEM (Hitachi S2400, Hitachi, Tokyo, Japan), after coating with gold-palladium alloy. The morphological characterization was complemented with pore size distribution measurement by MIP (AutoPore IV 9500 V1.09, Particle Technology Labs, Downers Grove, IL, USA). Samples with similar volume (approx. 0.3–0.5 cm^3^) were pre-conditioned at 40 °C, for 24 h, and kept under vacuum, before testing. The tests were carried out for mercury pressures up to 228 MPa, for an equilibrium time of 40 s. The pore size distribution was estimated considering a surface tension of 0.480 N/m at 20 °C and a contact angle of 140°, covering pore diameters as low as 7 nm. The XRD, ^29^Si-NMR and DSC/TG analyses of hardened samples were also carried out, following the procedures described in Section 2.3.1.

Similarly to the anhydrous cement powders, the hardened pastes were also characterized by TG. The analyses of thermal plots allowed to determine the percentage mass loss corresponding to dehydroxylation and decarbonation events taking place in hydrated powders with increasing temperature [34]. The hydration degree (*α_TG_*) of hydrated cement pastes was calculated using Equation (1).

(1) $$\alpha_{TG}=\frac{W_{b}}{W_{n}}\times 100$$

where *W_n_* is the weight fraction of water required for complete hydration of anhydrous cement (considered as 0.23 for ordinary PC [15]) and *W_b_* is the non-evaporable water, determined through Equation (2) [35].

(2) $$W_{b}=L_{dh}+L_{dx}+0.41\times \left(L_{dc}-L_{dca}\right)$$

where *L_dh_* is the mass loss corresponding to the dehydration of calcium silicate hydrates (C-S-H) and to the decomposition of gypsum and of aluminate phases (AFm and AFt), *L_dx_* is the mass loss corresponding to the dehydroxylation of CH, *L_dc_* is the mass loss corresponding to the decarbonation of carbonated phases and *L_dca_* is a correcting factor to account for the weight loss due to the decarbonation of filler and other carbonated materials in the anhydrous cement. The CH content was determined through Equation (3), where *L_dc__NT_* is a correcting factor that takes into account the decarbonation of carbonated materials in the OP and *M_CH_*, *M_H2O_* and *M_CO2_* correspond to the molecular weight of CH, H_2_O and CO_2_, respectively.

(3) $$CH=L_{dx}\times \frac{M_{CH}}{M_{H_{2}O}}+\left(L_{dc}-L_{dc_{NT}}\right)\times \frac{M_{CH}}{M_{CO_{2}}}$$

## 3. Results and Discussion

### 3.1. Characterization of the Produced Recycled Cements

#### 3.1.1. Morphology of Recycled Cements

**Figure 1:**
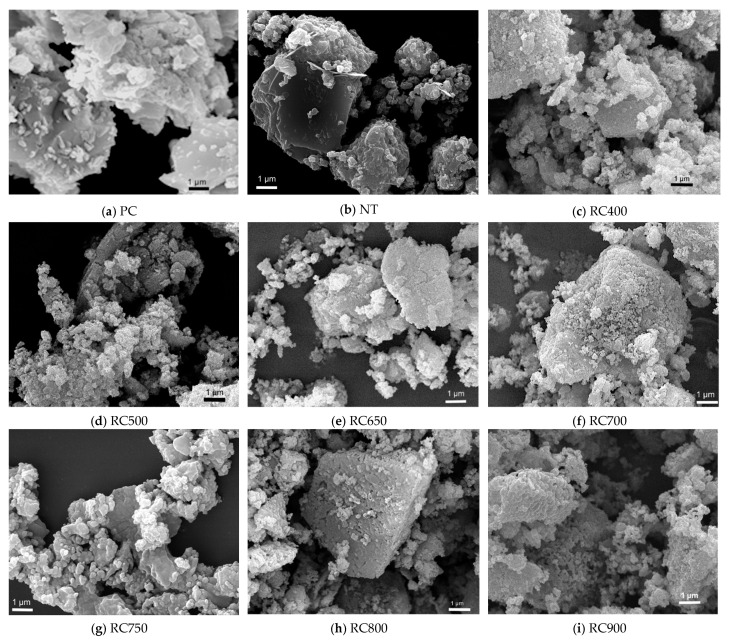
Cement particulates. FEG-SEM images of (**a**) PC, (**b**) NT waste cement, and RC treated at: (**c**) 400, (**d**) 500, (**e**) 650, (**f**) 700, (**g**) 750, (**h**) 800 and (**i**) 900 °C.

**Figure 2:**
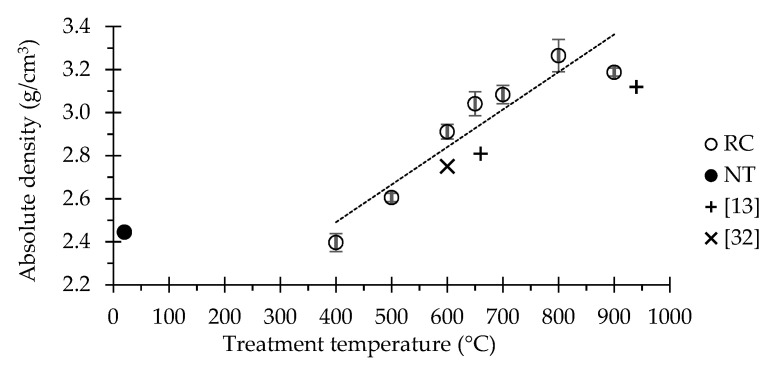
Measured absolute density of NT and of RC treated at different temperatures (results obtained by other authors also using helium pycnometry [14,36]).

Figure 1 presents the electron microscopy images of PC, NT and RC treated at different temperatures, and Figure 2 displays their absolute density.

**Figure 3:**
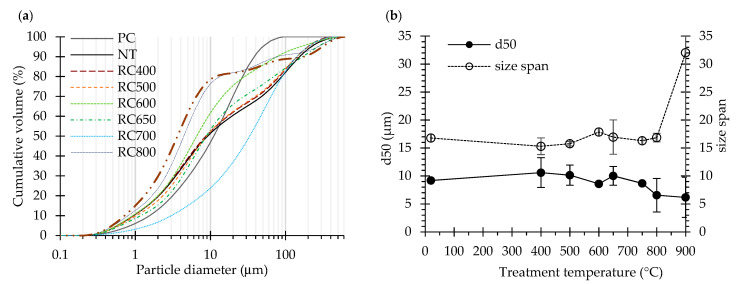
Particle size distribution of PC, NT and RC: (**a**) Cumulative volume distribution; (**b**) median particle size (d_50_), particle size span $\left(\frac{d_{90}-d_{10}}{d_{50}}\right)$.

The PC particles (Figure 1a) displayed an angular shape, mainly arranged in irregular loose agglomerates, while NT particles (Figure 1b) seemed to be less angular, with variable shape, ranging from thin plates to irregular spheres. Contrary to NT and RC particles, the PC grains showed a smooth surface and non-porous nature, as well as lower surface area for a given volume. In turn, the recycled cements (Figure 1c–i) displayed an apparently less angular and progressively rounder shape, while forming denser (Figure 2) and larger (Figure 3) arrangements with increasing treatment temperature. In fact, with increasing temperature and progressive dehydration, the granules became finer and rounder, apparently increasing their surface area. However, above 750 °C, a progressive binding of the finer granules seemed to have occurred, with a consequent reduction of the surface area.

The absolute density of NT powders (2.445 ± 0.042 g/cm^3^) increased with the treatment temperature up to 800 °C (3.265 ± 0.018 g/cm^3^) (Figure 2), reflecting the dehydration, dehydroxylation and decarbonation phenomena with consequent reduction of the specific volume, as well as a possible bonding promoted by adhesion between particles during calcination [37]. A further temperature increase to 900 °C resulted in a slight decrease of absolute density to 3.188 ± 0.059 g/cm^3^, probably because the increasing amount of CO_2_ released during the decarbonation progress with temperature provided pressure for particles’ expansion and bloating [38]. Although following a similar trend, the results obtained in the present study were slightly higher than those reported in the literature for the same test method [14,36]. This is expected to have resulted from differences in the experimental details, since, besides the treatment temperature, the absolute density of recycled cements is also affected by the calcination procedure (heating and cooling rate and residence time) and by the characteristics of the origin material.

Figure 3a presents the particle size distribution of PC, NT and RC. Overall, the particle size tended to reduce with the treatment temperature. A progressive reduction of the median particle size (*d*_50_) was observed with increasing treatment temperature, especially over 400 °C (Figure 3b). This may be attributed to the differential thermal expansion between different phases and to the shrinkage promoted by the dehydration and decarbonation of RC [14]. In particular, the decarbonation showed a relevant influence on the reduction of the particle size of RC treated at 800–900 °C.

For treatment temperatures up to 650 °C, the reduction of the particle size was similar to that of NT, with only a slight size reduction of the coarser particles after thermal treatment (Figure 3). Unexpectedly, RC700 diverged from the trend, showing a much coarser particle size distribution than NT (Figure 3a), which suggests an eventual particle agglomeration.

In general, above 400 °C, the particle size span continuously increased with increasing treatment temperature. This indicates that, during burning, simultaneous coarsening of the larger particles and reduction of the finer particles occurs. On the one hand, granulation and bonding phenomena promoted by heating in rotary kiln lead to particle agglomeration and coarsening [37]. On the other hand, particle expansion, fracture and deagglomeration resulting from differential thermal expansion in the course of solid state transformations and from gaseous phase release during progressive dehydration and dehydroxylation (mainly up to 700 °C) [15] and decarbonation (mainly up to 800 °C) [14] lead to size reduction [37,38].

#### 3.1.2. Thermogravimetry of Recycled Cements

**Figure 4:**
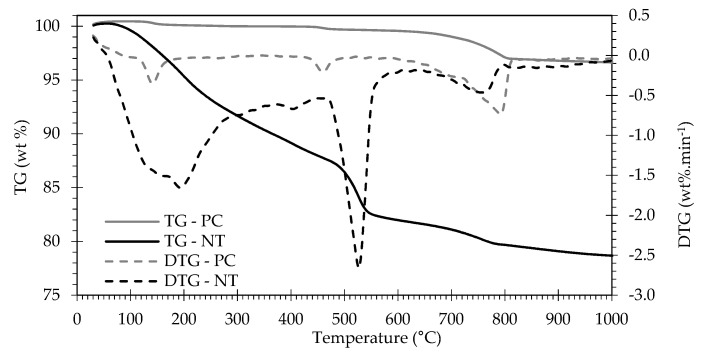
Thermogravimetric analysis: TG curves and first derivative (DTG) of PC and NT.

Figure 4 presents the TG and first derivative curves of PC and NT. The weight losses of PC are essentially related with gypsum decomposition, near 140 °C, and decarbonation of lime filler, at about 700–800 °C. The hydrated NT showed the typical weight loss areas related with the dehydration of C-S-H and decomposition of aluminate phases (*L_dh_* up to 470 °C), the CH dehydroxylation (*L_dx_* between 470–565 °C) and decomposition of carbonated compounds (*L_dc_* over 565 °C) [9,10,15,17,23,39].

**Figure 5:**
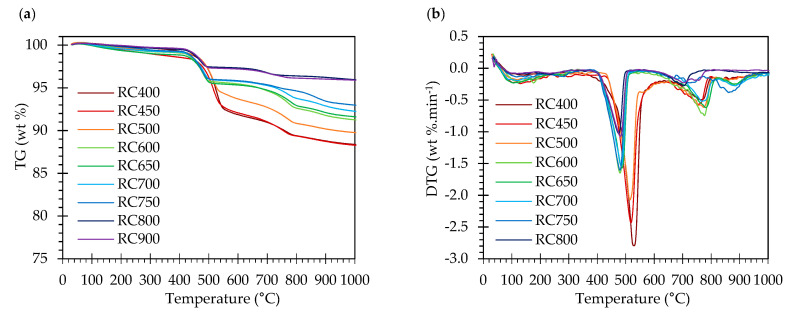
Thermogravimetric analysis: (**a**) TG curves and (**b**) first derivative (DTG) of RC treated at different temperatures.

Figure 5 displays the TG and first derivative curves of RC treated at temperatures between 400 and 900 °C. The corresponding weight loss values are rendered in Table 3.

Given that, during thermal activation, the RC had already been subjected to temperatures over 400 °C, the weight loss up to this temperature was not noteworthy. In the 400–500 °C range, RC treated up to 500 °C displayed significant weight loss, which is essentially due to the presence of residual CH from NT that was not dehydroxylated during thermal activation. In RC subjected to thermal treatment above 600 °C, the occurrence of dehydroxylation implies that hydration of CaO to form CH took place hitherto, either during cooling or storage. This phenomenon was less relevant in RC treated at 800 and 900 °C, possibly due to the lower surface area of the resulting RC (Figure 1h,i). Moreover, CH dehydroxylation extended throughout a wider temperature range (510–600 °C) in RC treated at 400–500 °C (Figure 5), suggesting that, in RC treated at higher temperatures, the newly formed CH (from CaO rehydration) had lower bonding energy, thus requiring lower temperature for dehydroxylation to occur. RC800 and RC900 did not present a significant weight loss, as would be expected, considering that, during thermal treatment, these RC had already been subjected to temperatures sufficiently high to promote decarbonation. Compared with NT, the amount of carbonated phases tended to increase slightly in RC (*L_dc_*, after taking into account the reduction of the hydration weight fraction in NT, Table 3), especially those treated above the dehydroxylation temperature. This indicates that some carbonation occurred after thermal treatment above 450 °C, as also suggested by Wang et al. [9] and Carriço et al. [22]. Overall, the CH content decreased and the estimated CaO content increased (Equation (4)) with the treatment temperature, as a result of CH dehydroxylation, as well as of CaCO_3_ decarbonation, depending on the treatment temperature (Table 3). The CaO content was estimated according to Equation (4), where *M_CaO_* is the molecular weight of CaO and the other variables were previously defined (Section 2.3.1).

(4) $$CaO=\;\left(L_{dx_{NT}}-L_{dx_{RC}}\right)\times \frac{M_{CaO}}{M_{H_{2}O}}+\left(L_{dc_{NT}}-L_{dc_{RC}}\right)\times \frac{M_{CaO}}{M_{CO_{2}}}$$

The calculated hydration degree (*α_TG_*) of NT was about 76 % (Equation (1)), which is consistent with a sufficiently hydrated paste, similar to that of a mature paste. As expected, the combined water in RC decreased with increasing treatment temperature, indicating the effectiveness of the thermal activation on the dehydration of NT. Nevertheless, the combined water (*W_b_*) in RC reached up to 4% for treatment temperatures over 600 °C, which may affect its rehydration activity.

#### 3.1.3. Effect of Treatment Temperature on the Phase Composition of Recycled Cements

**Figure 6:**
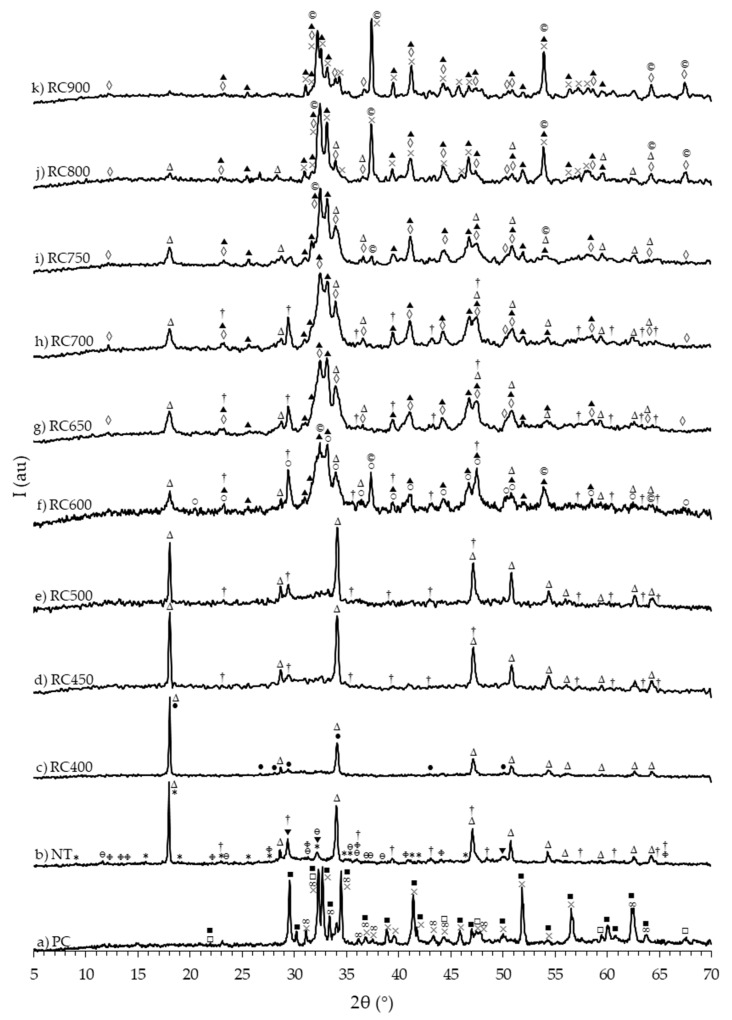
XRD analysis of PC, NT and RC. Δ CH (portlandite); † CaCO_3_ (calcite); * AF_t_ (ettringite); ∞ gypsum; © CaO; ■ C_3_S; ● tobermorite 9Å; ▼ C-S-H; × β-C_2_S; ▲ α’_L_-C_2_S; ○ C_3_A (calcium aluminum oxide); □ calcium aluminum iron oxide; ◊ C_4_AF (brownmillerite); Φ alumohydrocalcite; θ calcium aluminum oxide carbonate hydrate.

Figure 6 presents the XRD results of PC, NT and RC treated between 400 and 900 °C.

The diffractogram of PC (Figure 6a) displayed the main diffraction peaks of the crystalline phases usually present in clinker, including larnite (β-C_2_S), alite (C_3_S) and gypsum. The diffractogram of NT (Figure 6b) exhibited peaks corresponding to diffraction by CH, C-S-H and ettringite (phases commonly found in hydrated PC pastes), as well as calcite and other carbonated aluminate peaks, expectedly corresponding to the carbonation of the paste during milling and/or storage.

Although the full dehydroxylation of CH in RC would be expected above around 600 °C, with the decrease in the number and intensity of CH diffraction peaks and the corresponding increase of CaO peaks, this was not verified in any of the powders. Even though a major decrease in peak intensity took place at 600 °C (Figure 6f) and especially above 800 °C (Figure 6j), CH diffraction peaks were identifiable at all tested temperatures. This is in good agreement with the TG results (Figure 5, Table 3), and is expected to have resulted from the high pre-hydration susceptibility of RC. Furthermore, essentially due to the fact that NT was carbonated, calcite peaks were also identified in all RC, although a major decrease occurred above 750 °C, accompanied by the respective increase of CaO peaks.

The XRD analyses of RC treated at 400 (Figure 6c), 450 (Figure 6d) and 500 °C (Figure 6e) confirmed the partial decomposition of hydrated products, with the dehydration of C-S-H to tobermorite 9 Å and the absence of ettringite (within the diffractometer detection limit). At 450 and 500 °C, incipient C_2_S peaks, corresponding to the four most intense diffraction planes were detected, suggesting the onset of C-S-H depolymerization with α’_L_-C_2_S crystallization. In RC treated at 600 °C (Figure 6f), the diffraction profile changed considerably, with the presence of C_2_S peaks in increasing number and intensity and the disappearance of tobermorite 9 Å peaks (within the diffractometer detection limit). The full C-S-H depolymerization was thus expected at this temperature, although the noise associated to the plot and the large width of the most intense C_2_S peaks (in the 2θ range between 30° and 35°) indicated that the phase was not fully crystallized. Moreover, although the peak positions and relative intensities of α’_L-_C_2_S and α’_H-_C_2_S diffractograms are very similar (ICCD files 31-0299 and 31-0298, respectively), the presence of the low intensity peaks in the 53.5–56° 2θ range suggests that the polymorph present in RC treated at 650 (Figure 6g), 700 (Figure 6h) and 750 °C (Figure 6i) was α’_L-_C_2_S. At 800 (Figure 6j) and 900 °C (Figure 6k), the α’_L-_C_2_S peaks were more intense and well-defined, indicating a higher crystallinity, in line with the literature [9,12,14]. At these temperatures (800–900 °C), the β-C_2_S polymorph was also identifiable. This indicates that, at high treatment temperatures, less reactive calcium silicate polymorphs are formed [12,14].

**Figure 7:**
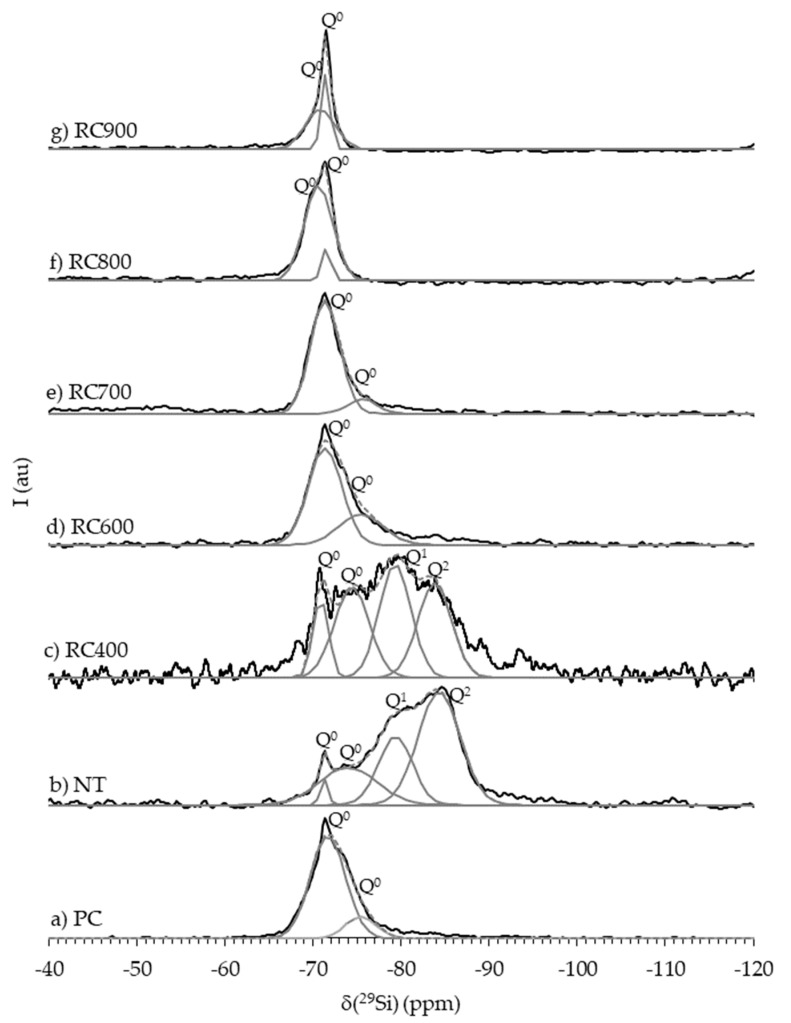
^29^Si-NMR spectra of PC, NT and RC thermoactivated in the 400–900 °C temperature range (peak deconvolution is displayed in gray). Q^n^ refers to the number of orthosilicate units (*n*) attached to a SiO_4_ tetrahedron (*Q*).

The ^29^Si-NMR spectra of PC, NT and RC treated between 400 and 900 °C (Figure 7) rendered additional information regarding the evolution of cement structure and reaction state with increasing treatment temperature.

The deconvolution of the ^29^Si-NMR spectrum of PC (Figure 7a) showed two Q^0^ peaks at δ(^29^Si) of −71.67 and −75.40 ppm (Table 4), suggesting that only monomeric orthosilicates, typical of anhydrous PC, were present [10,40,41]. The XRD analysis results (Figure 6a) indicated that these calcium silicates corresponded β-C_2_S and C_3_S. Four peaks were identified in the ^29^Si-NMR spectrum of NT (Figure 7b), at δ(^29^Si) of −71.27 and −73.98 (Q^0^), −79.33 (Q^1^) and -83.80 ppm (Q^2^) (Table 4). The Q^0^ resonances in NT indicated that part of the calcium silicates from PC remained unreacted, although the decrease in peak intensity suggested that these were less abundant than in PC. The Q^0^ peak at a δ(^29^Si) of −71.3 ppm can be attributed to β-C_2_S [40,41,42]. Since this polymorph is known to react slowly over time in PC pastes, a low amount was expected to be present even at high temperature, mixed with newly formed C_2_S polymorphs [10]. However, β-C_2_S was not clearly detected by the XRD analysis of NT (Figure 6b) nor of RC treated up to 800 °C (Figure 6c–e), suggesting that the amount of this anhydrous compound was below the diffractometer detection limit. The presence of Q^1^ and Q^2^ refers to the resonance of an end-chain group and of a middle chain group, respectively, corresponding to the polymerization of C-S-H with increasing gel chain length in the course of the hydration process [43].

The comparison between the spectra of NT and of RC treated at increasing temperatures confirmed the gradual depolymerization of C-S-H. The spectrum of RC400 (Figure 7c) was relatively similar to that of NT, also displaying four main resonance peaks (Table 4) at δ(^29^Si) of −70.86 and −74.40 (Q^0^), −79.33 (Q^1^) and −83.80 (Q^2^) ppm. The decrease of the Q^2^/Q^1^ ratio (Table 4) suggests the decomposition of C–S–H gel, rendering a shorter chain length compared to that of NT. Moreover, the Q^0^ peaks presented approximately the same δ(^29^Si), but were narrower than in NT, indicating that monomeric orthosilicates were more confined in the overall cement structure. The ^29^Si-NMR profiles were considerably different in RC treated at higher temperatures.

Above 600 °C, the Q^1^ and Q^2^ peaks were no longer identifiable, confirming the full depolymerization of C-S-H and the formation of anhydrous calcium silicates. Similar findings were obtained by Lü et al. [12], in RC treated above 650 °C. The ^29^Si-NMR spectra of RC600 (Figure 7d) and RC700 (Figure 7e) displayed a main Q^0^ peak at a δ(^29^Si) of −71.35 ppm. Although this is usually attributed to anhydrous β-C_2_S [40,41,44,45], in the 600–750 °C range, the only C_2_S polymorph detected in the XRD analysis within the diffractometer limit was α’_L_-C_2_S (Figure 6f–h). Furthermore, Hong and Young [46] demonstrated that α’_L_-C_2_S synthesized from C_2_S calcined at 700 °C displayed the main resonance peak at a δ(^29^Si) of −71 ppm, and could thus be mistaken with the typical single peak of β-C_2_S, because the δ(^29^Si) of calcium silicates containing Q^0^ units depend strongly on their formation and stabilization conditions [44]. Taking this into account, this resonance was attributed to α’_L_-C_2_S.

At 800 °C, a new different Q^0^ resonance emerged at a δ(^29^Si) of −70.60 ppm (Figure 7f), which is consistent with the progressive formation of β-C_2_S [11,22,44] identified in the XRD analysis (Figure 6j). At 900 °C, this resonance shifts to a δ(^29^Si) of −70.81 ppm (Figure 7g), narrower and more intense than at 800 °C. Overall, the Q^0^ peaks gradually became sharper with increasing treatment temperature, indicating higher crystallinity, which is consistent with the results of the XRD analysis.

### 3.2. Hydration of Recycled Cement Pastes

#### 3.2.1. Water Demand and Setting Time of Recycled Cement Pastes

**Figure 8:**
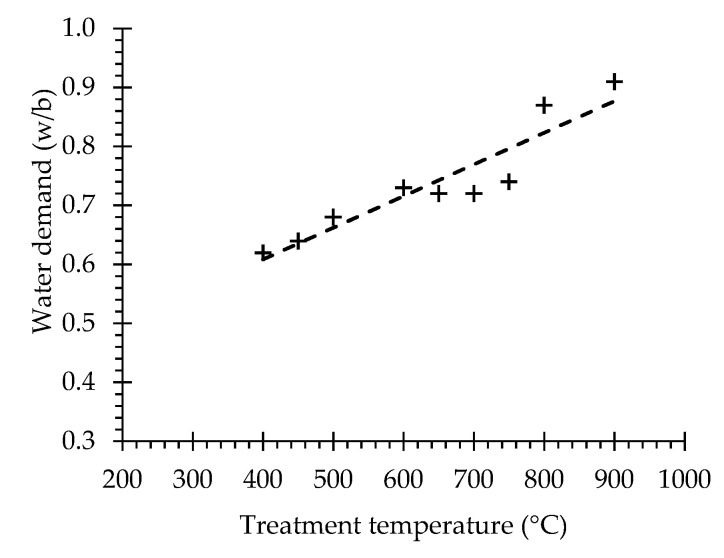
Water demand of RC treated at different temperatures.

**Figure 9:**
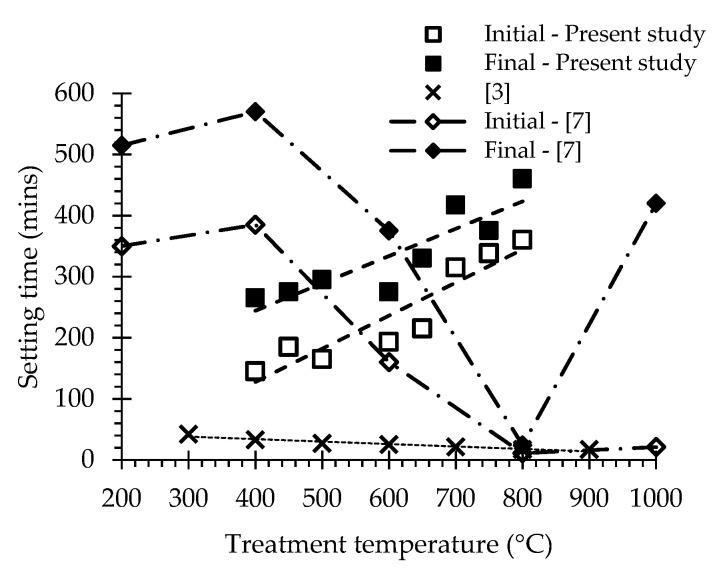
Setting time of RC treated at different temperatures. Values reported by other authors shown for comparison [4,8].

The water demand (Figure 8) and setting time (Figure 9) results of PC and with RC treated at different temperatures are summarized in Table 5. While the water demand for normal consistency of the PC paste corresponded to a w/b of 0.31, that of the RC pastes ranged from 0.62 to 0.91 (which is about 3-fold that of the PC paste) (Figure 8), increasing with the treatment temperature. This is in line with the results reported by other authors [4,8,9,13,23]. According to Shui et al. [4], this is due to the increase of the surface area of dehydrated phases and the formation of free CaO in recycled cements. In fact, the CaO present in RC immediately reacts with part of the mixing water, while another part evaporates due to the exothermic nature of that reaction. This suggests that the more significant increase of water demand of RC treated at 800 and 900 °C is associated to higher free CaO content (Table 3), as well as to their higher fineness, compared to RC treated at lower temperatures (Figure 3). The different fineness values also explain the slightly higher water demand of RC600 compared to RC650–RC700 (Figure 3). Another contribution to the higher water demand is expected to arise from increased water absorption, due to the porous nature of dehydrated RC particles [5,6,23].

The RC treated at 400 °C to 800 °C presented initial setting times between 145 and 360 min and final setting times between 265 and 460 min. These setting time values were at least about twice as high as those of PC and tended to increase with the treatment temperature (Figure 9), contrarily to the general tendency reported in the literature [4,8,13,15]. Usually, setting times faster than those of PC are reported, being ascribed to the fast rehydration of RC, due to their high reactivity and surface area [4,5,6]. Moreover, the reduction of the setting time with increasing treatment temperature up to 800 °C has also been reported [4,5,6] (Figure 9). Vyšvaril et al. [8] associated the decrease of the setting time with the increase of CaO content with the treatment temperature. Serpell and Lopez [14] further stated that the CaO content is also responsible for an early “false setting” phenomenon. As mentioned, the precocious hydration of CaO in RC was detected in the present study (Section 3.1.2), which may have partly hindered the setting of RC pastes. Moreover, the particle size of RC (up to 250 µm, Section 3.1.1) was also higher than that reported in other studies (75 µm [4,14], 100 µm [6,11], 125 µm [8], 150 µm [5,9]). Above 800 °C, the setting time significantly increased, due to the loss of RC reactivity (Section 3.2.2) and to particle binding (Figure 1, Section 3.1.1).

The flowability of the pastes did not vary significantly with the treatment temperature (Table 5), since all pastes targeted normal consistency by changing the w/b, except PC1. PC1 was a fluid paste, since it was prepared with the same w/b as P700 (0.72). Yet, RC700 presented significantly lower flowability than PC1, which, as mentioned, is expected to have resulted from the higher surface area and higher absorption ability of RC700 compared with PC. Indeed, the replacement of RC with PC allowed to move from pastes of plastic consistency to self-compacting grouts.

#### 3.2.2. Hydration Heat of Recycled Cement Pastes

**Figure 10:**
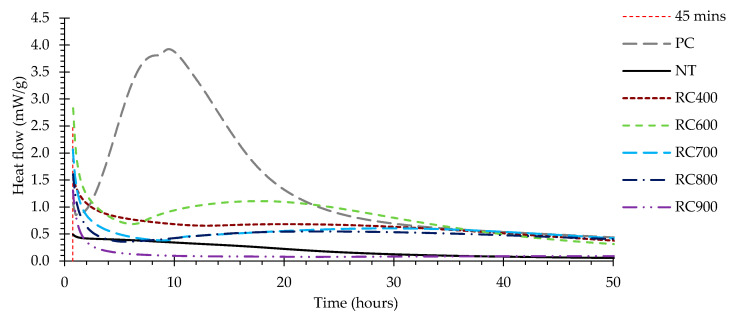
Heat flow over time of pastes produced with PC, NT and RC, during the first 50 h of hydration. Pastes with a w/b of 1.0.

**Figure 11:**
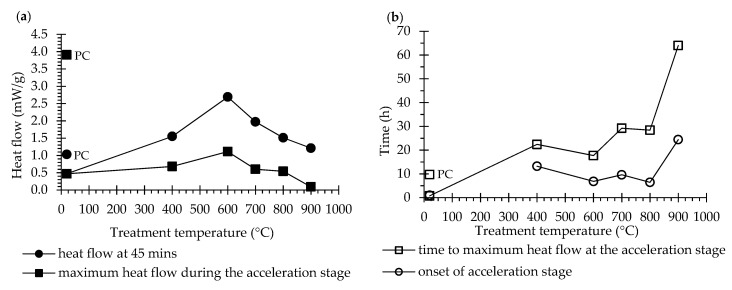
Features of the isothermal calorimetry curves, obtained throughout the hydration process of PC, NT and RC pastes. (**a**) Heat flow: at 45 min (the beginning of the calorimetric register) and maximum during the acceleration stage; (**b**) elapsed hydration time until onset of the acceleration stage and until maximum heat flow at this stage.

Figure 10 presents the heat flow over time of pastes produced with different RC and with PC and a w/b of 1.0. The hydration heat release curves of PC, NT and RC pastes were significantly different, especially in the first 30 h after mixing (Figure 10). The heat release could not be measured during the first 45 min, because only external ampoules were available. Nevertheless, the trend during the initial hydrolysis stage can be established from comparison of the obtained curves at 45 min: the heat release of the RC pastes ranged from 1.55 mW/g (paste with RC400) to 2.69 mW/g (paste with RC600), and tended to decrease with increasing treatment temperature down to a minimum of 1.21 mW/g (paste with RC900) (Figure 11). These values were higher than the hydration heat released by the PC paste (1.03 mW/g), which was also found by other authors [5,6,15,26]. In fact, Zhang et al. [6] reported an initial heat release value in paste with RC treated at 600 °C about 10 times higher than that of PC paste. A similar difference was observed by Baldusco et al. [5], in pastes with RC treated at 500 °C. The high surface area of RC and the instability of its dehydrated products, likely to quickly re-polymerize [4], together with the high calcium aluminate content of RC [5] and the highly exothermic CaO hydration reaction [9,15,26] have been presented as possible justifications for the higher initial hydration heat.

The shape of the remaining hydration heat rate curve was similar in RC and PC pastes, presenting induction, acceleration and deceleration stages. However, while the PC paste required about 0.9 h for acceleration stage onset and around 9.7 h to reach the maximum heat flow, the RC pastes demanded significantly longer periods (Figure 10). The pastes produced with RC600 (6.8 h) and RC900 (24.4 h) required the shortest and longest time until acceleration onset, respectively. These pastes also required shortest and longest time to reach the acceleration peak, respectively (17.7 h in RC600 and 64.4 h in RC900, Figure 10). Additionally, the slope of the calorimetric curves also showed that during the acceleration stage, the heat release in the RC pastes proceeded at much slower rate than in the PC paste (Figure 10). The maximum heat flow during the acceleration stage of the PC paste (3.91 mW/g) was much higher than that of any of the RC pastes, which increased from 0.68 mW/g, in the paste with RC400, up to 1.1 mW/g, in the paste with RC600, and decreased in pastes with RC treated at temperatures above 600 °C (Figure 11). This trend is also in line with the literature [5,9,15,26].

The highest RC hydration heat release was reached by the paste with RC600 and then decreased with increasing treatment temperature (Figure 11). At this range of treatment temperatures, upon contact with water, the free CaO and calcium aluminates quickly react and the newly formed C_2_S polymorph hydrates. Since the CaO content increased with the treatment temperature (Table 3), the high initial hydration heat cannot be entirely attributed to its hydration. The reactivity of newly formed compounds may have also contributed to this.

Neither the paste with RC400 nor the paste with RC900 were able to develop significant hydration. The paste produced with RC400 presented a very shallow acceleration stage and insignificant long-term reactivity, given that its hydration products were only partly dehydrated, without significant depolymerization (Section 3.1.3). The high initial heat release of the paste with RC400 can be attributed to the rehydration of C-S-H upon contact with water, arguing that the involved mechanism was an instantaneous rehydration of the dehydrated cement paste, rather than a dissolution-precipitation mechanism observable in the hydration of PC. In turn, the low hydration ability of the paste with RC900 is expected to have resulted from the lower reactivity of the composing calcium silicate compounds, as found in the XRD and ^29^Si-NMR analyses (Figure 6, Table 4). Overall, the RC pastes tended to present longer induction, acceleration and deceleration periods with lower hydration heat release than the PC paste (Figure 11), and although they displayed higher initial hydration heat, they developed less long-term hydration reactions than the PC paste.

Finally, NT showed only minor reactivity, since it was already sufficiently hydrated before mixing. The residual hydration heat release due to the reaction of remaining anhydrous compounds was not significant. The same was observed by Angulo et al. [26].

### 3.3. Properties of Hardened Pastes Prepared with Recycled Cement

Table 6 summarizes the hardened density and mechanical properties of the produced recycled cement pastes. The density values of hardened RC pastes tended to decrease with increasing treatment temperature, due to the higher w/b required to achieve the target normal consistency. The hardened density was similar in PC1 and P700 of equal w/b (0.72), confirming the similar particle density of the respective binders. The reaction of CaO with water, with water consumption and evaporation, is expected to have only a slight effect on the increase of paste density.

#### 3.3.1. Mechanical Strength of Recycled Cement Pastes

**Figure 12:**
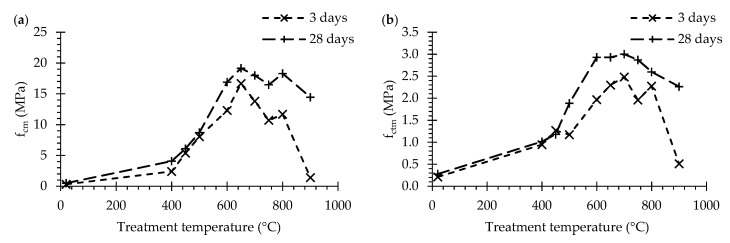
Mechanical properties of RC pastes at 3 and 28 days: (**a**) compressive strength, *f_cm_*; (**b**) flexural strength, *f_ctm_*.

Figure 12a,b refer to the compressive and flexural strength, respectively, at 3 and at 28 days of pastes produced with RC treated at different temperatures. As would be expected, the strength values were higher at 28 than at 3 days, regardless of the treatment temperature. The compressive strength values at 28 days ranged from 4.1 MPa (P400) up to a maximum of 19.2 MPa for P650, having decreased for pastes with RC treated at higher temperature (Table 6). The flexural strength values at 28 days ranged from 1.0 (P400) up to 3.0 MPa for P700, having decreased for pastes with RC treated at higher temperature (Table 6). In turn, PNT displayed only residual values of compressive (0.5 MPa) and flexural (0.3 MPa) strength when compared to the RC pastes, confirming that, without thermal activation, waste cement has insignificant rehydration ability. The residual mechanical strength developed by PNT can be attributed to the formation of hydration products from remaining unhydrated PC particles in NT.

**Figure 13:**
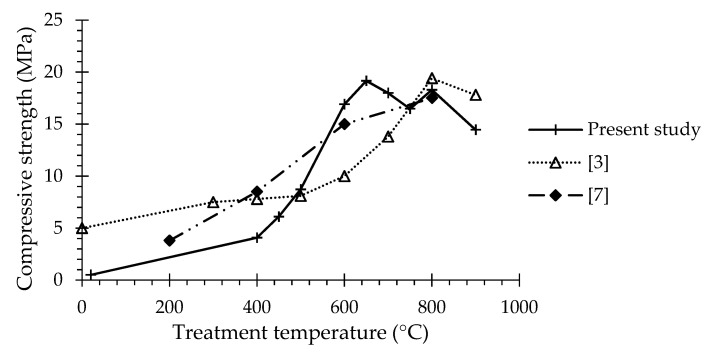
Compressive strength of RC pastes at 28 days compared with the results of other authors [4,8].

In spite of the increment of the w/b, the mechanical strength of RC pastes of similar consistency increased with RC treatment temperature up to 650 °C (Table 6, Figure 12). Similar trends were reported in the literature [4,8,13] (Figure 13). For treatment temperatures up to 500 °C, low mechanical strength values were achieved, expectedly because RC particles were only partially dehydrated and not totally depolymerized (Section 3.1.3). Therefore, the rehydration occurred in an already formed structure, not contributing to the global increase of cohesion (i.e., no significant additional links were established between anhydrous particles) nor to the mechanical strength of the bulk cement paste. A relevant improvement of mechanical strength took place after the dehydration phase, which is associated with the progressive morphological change of RC and with the formation of new anhydrous compounds.

On the other hand, treatment temperatures above 600 °C led to the progressive depolymerization of the RC (Figure 7), which were expected to display fast rehydration capacity (Section 3.2.2). For treatment temperatures above 700 °C, the compressive and flexural strengths of RC pastes with the same consistency tended to decrease with the treatment temperature. This is expected to have resulted from the lower specific area of RC particles and lower reactivity of C_2_S polymorphs above this temperature, as discussed in previous sections. This was particularly evident for RC treated at 900 °C, in which the mechanical strength at 3 days was about 10 times lower than that of other pastes with RC treated over 600 °C, including that of P800 with similar w/b.

Overall, the increase of mechanical strength with age tended to be more relevant for RC pastes treated above 700 °C (Figure 12), in line with the calcium silicate phases of lower reactivity identified above this treatment temperature (Figure 6).

**Figure 14:**
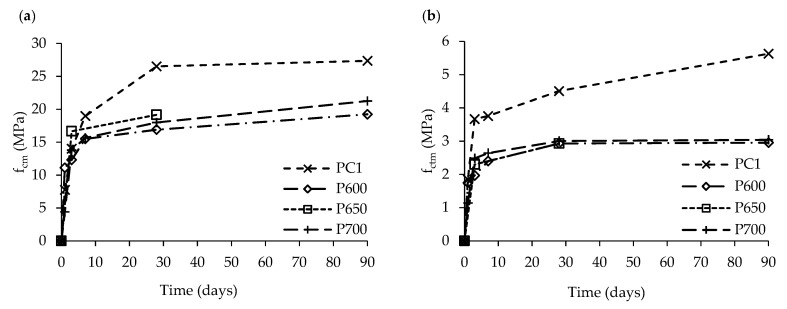
Mechanical properties of PC1, P600, P650 and P700 over time: (**a**) compressive strength, *f_cm_*; and (**b**) flexural strength, *f_ctm_*.

Figure 14 compares the compressive (Figure 14a) and flexural (Figure 14b) strength over time of PC1, P600, P650 and P700 with similar w/b (Table 6). While the compressive strength evolution of P600-P700 was similar to that of PC1 up to 3 days, it was lower from then on and, at 28 days, its value reached about 73% of that of PC1 (Figure 14a). A similar behavior was observed regarding flexural strength (Figure 14b). According to the literature, the fast initial strength development of P600–700 can be attributed to the high surface area of RC particles [4,5,15]. Moreover, part of the mixing water is expected to be initially consumed on the formation of internal hydration products within the porous microstructure of RC pastes, leaving less water available between anhydrous particles. Therefore, the bulk RC paste behaves as having a lower w/b and the proximity between particles increases, which accelerates the cohesion between them and improves the early strength. This is further discussed in Section 3.3.2.

As shown in Table 6, the compressive strength of RC600 was 42% higher than that of PC1 at 1 day. However, at later ages, more external hydration products can be formed in non-porous PC pastes, inverting the trend, as occurred after 3 days. Furthermore, the porous RC pastes seem to be associated with a strength threshold (Figure 14), where significant strength development over time is prevented [6].

The mechanical strength of PC2 was up to 6 times higher than those of RC pastes of the same consistency (Table 6), independently of the treatment temperature. This was essentially attributed to its significantly lower w/b (0.31), when compared with those of RC pastes.

#### 3.3.2. Microstructural Characterization of Hydrated Recycled Cement Pastes

**Figure 15:**
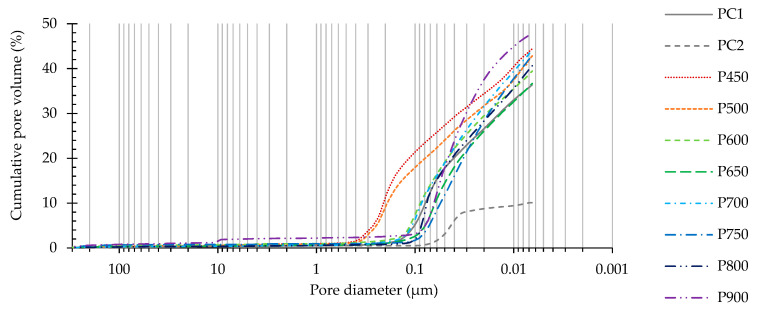
Cumulative pore volume as a function of pore diameter of PC1, PC2 and RC pastes at 28 days.

**Figure 16:**
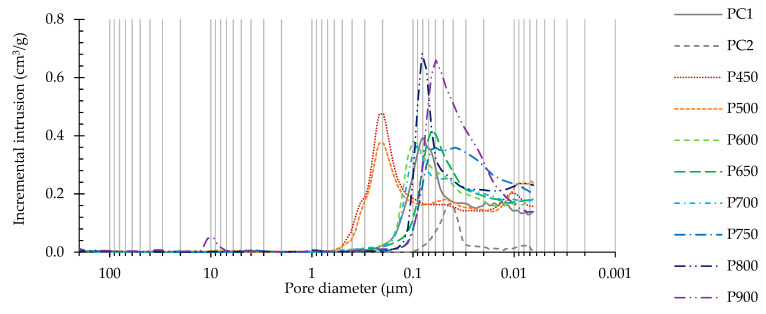
Incremental intrusion as a function of pore diameter of PC1, PC2 and RC pastes at 28 days.

Figure 15 and Figure 16 present the cumulative pore volume and the incremental intrusion as a function of the pore diameter, and Table 7 displays the corresponding total porosity and critical pore diameter of PC and RC pastes at 28 days, respectively. Note that pores between C–S–H gel particles (gel pores, with typical diameter between about 0.5–5 nm [47]) are outside the MIP measuring range (Section 2.3 [48]). In all produced pastes, the pore size distribution was continuous, ranging approximately from 0.007 to 45 µm, i.e., in the capillary pores range [47].

As well known, the w/b ratio is the main factor affecting the microstructure porosity. Therefore, the PC2 paste produced with a w/b of only 0.31 had the lowest total porosity, having been about 26–34% of that of RC pastes of identical consistency (Table 7), but with much higher w/b ratio (0.64–0.91). Compared to PC1 of equal w/b (0.72), the RC pastes (P600–P750) showed similar porosities, varying between 37.49% and 42.79%, depending on the treatment temperature (Table 7).

**Figure 17:**
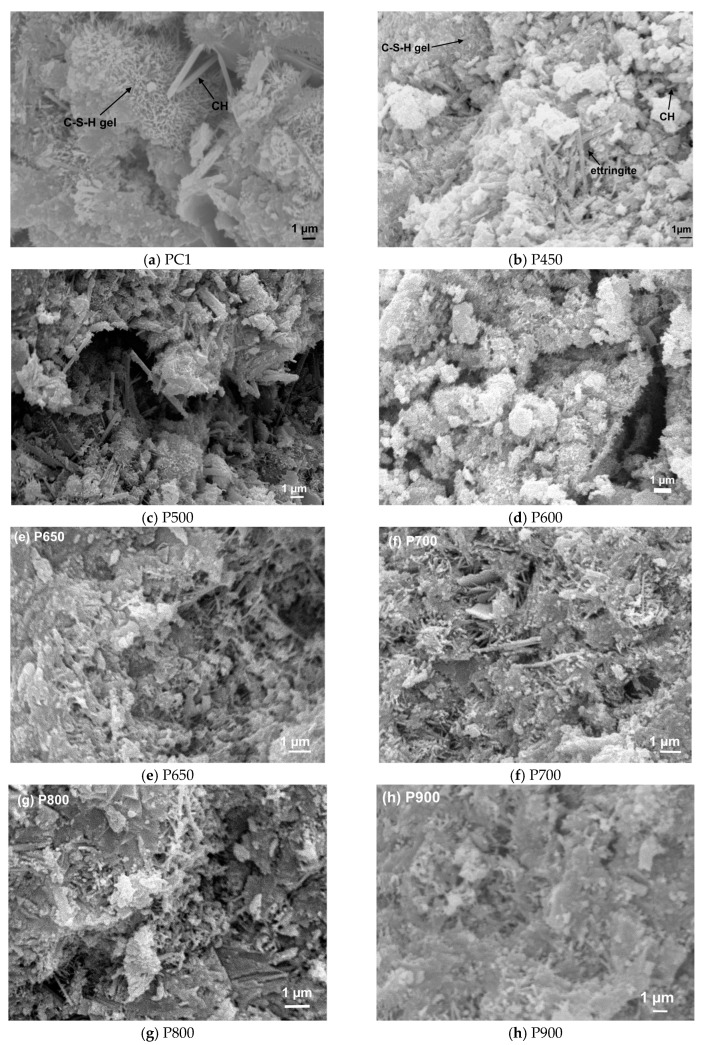
SEM images of pastes at 28 days: (**a**) PC1; (**b**) P450; (**c**) P500; (**d**) P600; (**e**) P650; (**f**) P700; (**g**) P800 and (**h**) P900.

Despite their lower w/b, pastes with RC treated up to 500 °C (Figure 17b,c) presented coarser porosity than PC1 (Figure 17a) and other RC pastes (Figure 17d–g), displaying the highest critical pore diameter value. This is expected to have resulted from the poor development of external hydration products outside the RC particles, leading to a loose microstructure of high open porosity (Figure 17c). Therefore, the cumulative pore volume and pore size distribution curves in these RC pastes were skewed in the coarser porosity direction. As discussed in Section 3.2.2, the setting of RC treated up to 500 °C is based on a simple rehydration mechanism, without contributing for the significant development of new external repolymerization products. However, the total porosity of such pastes was only slightly higher than those of PC1 and other RC pastes (Table 7). This could have resulted from the fact that part of the mixing water was consumed in the internal rehydration of RC particles, conserving the same microstructure of the origin NT. On the other hand, pastes produced with RC treated above 600 °C (Figure 17d–g) displayed a more refined porosity than PC1 of similar w/b, even when the total porosity was higher (e.g., P750).

**Figure 18:**
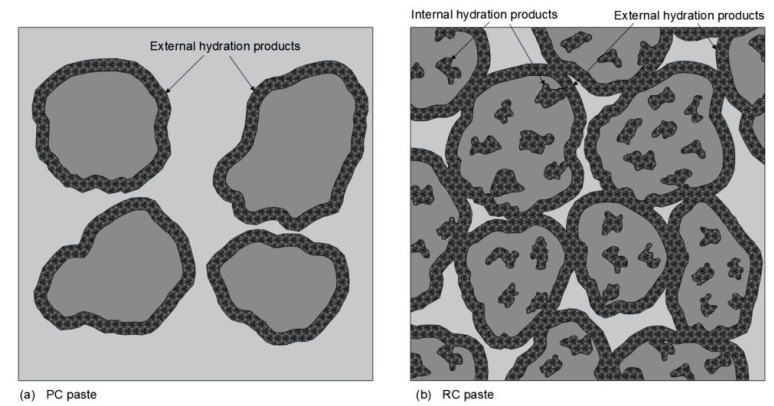
Schematic representation of microstructure development in PC and RC pastes.

In fact, contrary to common PC pastes, RC pastes are expected to develop two distinct microstructures, one within the porous RC particles (internal products) and other concerning the surrounding bulk paste (external products). This is schematically presented in Figure 18.

Since part of the water was consumed in the development of internal products, the w/b of the external bulk paste was reduced, and the particles became closer. Therefore, the external porosity was finer and the cumulative pore volume and pore size distribution were skewed to the finer size of the distribution, approaching pastes of lower w/b (PC2). However, since RC particles are porous, the mercury intrusion volume increased significantly after crossing this protective paste barrier, and the total porosity became equivalent to that of the PC paste of the same w/b (PC1). Overall, the main conclusion that may be drawn is that the total porosity of pastes with RC treated over 600 °C depends mainly on the w/b, similarly to PC pastes. Basically, for the same total porosity volume (equal w/b), the microstructure of PC pastes should be coarser than that of RC pastes, which are able to distribute the available water outside and inside their porous particles.

The lowest total porosity value was achieved for P650, which is in accordance with the higher mechanical strength displayed by this paste (Table 7). This paste showed quicker reactivity, developing denser external structures. The difference of external microstructure between RC and PC pastes is expected to be more relevant at early ages (as discussed in Section 3.3.1) and tend to be diluted at later ages, when the increase of external hydration products in PC pastes compensates the initially larger available space between their particles. On the other hand, after the initial hydration period, the development of external products in RC pastes is conditioned by those already developed within the particles. The amount of external products developed in PC pastes is thus expected to be higher than in RC pastes. However, the space available between particles at the beginning of the hydration process is also higher in PC pastes. The coarser and higher porosity of P900 compared to that of other RC pastes can be explained by its higher w/b and slower reactivity.

Furthermore, the morphological analysis of pastes with 28 days (Figure 17) supports the obtained porosity results, and, thus, the mechanical performance. Similarly to the PC paste with equal w/b (PC1, Figure 17a), RC pastes (Figure 17b–h) presented evidence of rehydration, displaying common hydration products that include ettringite needles, clusters of C-S-H gel and CH hexagonal platelets. However, different microstructural features were observed depending on the treatment temperature of the corresponding RC. Up to 500 °C, the RC pastes showed loose microstructure, where anhydrous particles were easily identifiable, even after 28 days. The lack of cohesion was observable, confirming the low depolymerization and formation of new external products. Large CH crystals (also present in the original NT cement), ettringite needles and surface C-S-H gel were dispersed in an open porous structure. This observation is in line with the MIP results (Figure 15) and justifies the low mechanical strength achieved in these pastes (Figure 12). Since part of the mixing water was absorbed and consumed by RC particles, the external products developed in a matrix with a lower w/b are characterized by a higher proximity between anhydrous RC particles than in PC1 of similar w/b. This may explain the slightly coarser porous structure observed in the PC paste (Figure 17a), as also identified in the MIP results (Figure 15). Nevertheless, a higher volume of external hydration products seems to have developed in the PC pastes at 28 days, which justifies the higher mechanical strength than that of RC pastes. On the other hand, above 600 °C, the RC pastes were able to develop new hydration products, that resulted in the refinement of the pore structure (Figure 15). Moreover, the RC pastes displayed an apparently rougher surface than PC1, which can be attributed to the higher surface area of anhydrous particles and to the faster hydration of RC.

The mechanical strength results, supported by the microstructural analysis, suggest that the optimal treatment temperature, regarding the best technical, environmental and economical compromise, is about 600–650 °C. Above this temperature, an eventual slight improvement of the mechanical strength should not compensate the increase of energy consumption and greenhouse gas emissions.

#### 3.3.3. Thermogravimetry of Hydrated Recycled Cement Pastes

**Figure 19:**
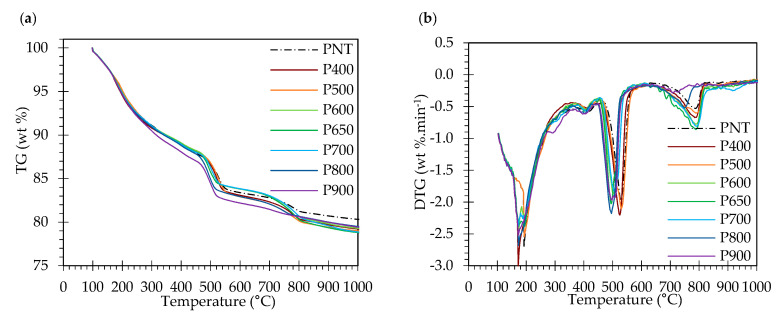
Thermogravimetric analysis: (**a**) TG curves and (**b**) first derivatives (DTG) of PNT and RC pastes at 28 days.

**Figure 20:**
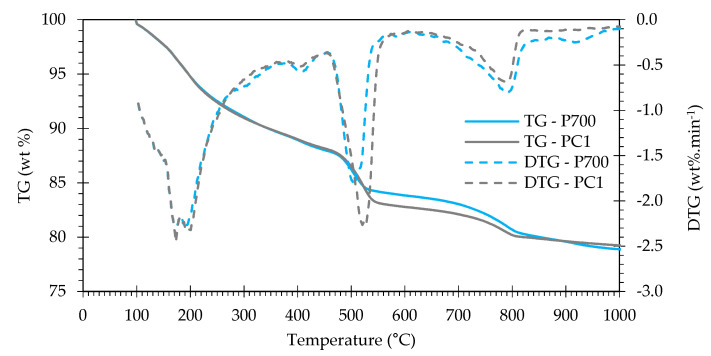
Thermogravimetric (TG) and first derivative (DTG) analysis of PC1 and P700 at 28 days.

Figure 19 presents the TG and first derivative curves of the produced pastes, at 28 days. The RC pastes presented a similar weight loss (Figure 19) up to the dehydroxylation stage (*L_dh_*, Table 8), indicating that the rehydration was effective, regardless of the treatment temperature. Moreover, the fraction of binding water was similar to that of the PC paste of equal w/b, suggesting the formation of a comparable amount of hydration products (Figure 20, Table 8). However, as discussed in Section 3.3.1, the rehydration of non-depolymerized RC treated up to 500 °C did not promote a significant formation of strong cohesion bonds between anhydrous particles. The internal products developed within the porous RC particles did not contribute to the cohesion of the bulk cement paste. The weight loss related to CH dehydroxylation occurred earlier in pastes with RC treated above 600 °C than in NT and P400–P500 (Figure 19), which is expected to have resulted from the lower binding energy associated to the newly formed CH.

Except for P800 and P900, which were produced with fully decarbonated RC, all other RC pastes displayed a considerable weight loss above 600 °C, having been higher than that of NT (Table 8). Furthermore, the decarbonation stage tended to end at higher temperatures in RC pastes than in NT (except for P800 and P900). Noteworthy is the fact that the additional carbonation not accounted for in the anhydrous RC tended to increase with the treatment temperature. Given that all pastes were cured in water until hydration arrest with isopropanol, further carbonation should not have occurred during the curing period. This phenomenon can be ascribed to the formation of CaCO_3_ through the reaction of CH with the organic solvents released during this procedure [49].

Despite small differences, the TG curves of P700 and PC1 (of equal w/b) showed a similar development, suggesting the formation of rehydration products of identical type and morphology (Figure 5). Differences between the curves at about 270–330 °C may be related to the formation of higher amounts of carbonate or sulfate AFm phases in RC [49]. Furthermore, P700 presented a lower weight loss due to CH dehydroxylation, but a higher weight loss due to decarbonation (Table 8). Even considering the higher carbonation of RC, lower CH was developed in RC pastes than in PC pastes (Table 8). The apparent higher hydration degree (α_TG_) of pastes with RC treated above 700 °C is related to the higher availability of CH from calcite decarbonation (Table 8). In this case, one cannot consider that a higher hydration degree was attained in these pastes, as shown in ^29^Si-NMR analysis (Section 3.3.5).

#### 3.3.4. X-ray Diffraction of Hydrated Recycled Cement Pastes

**Figure 21:**
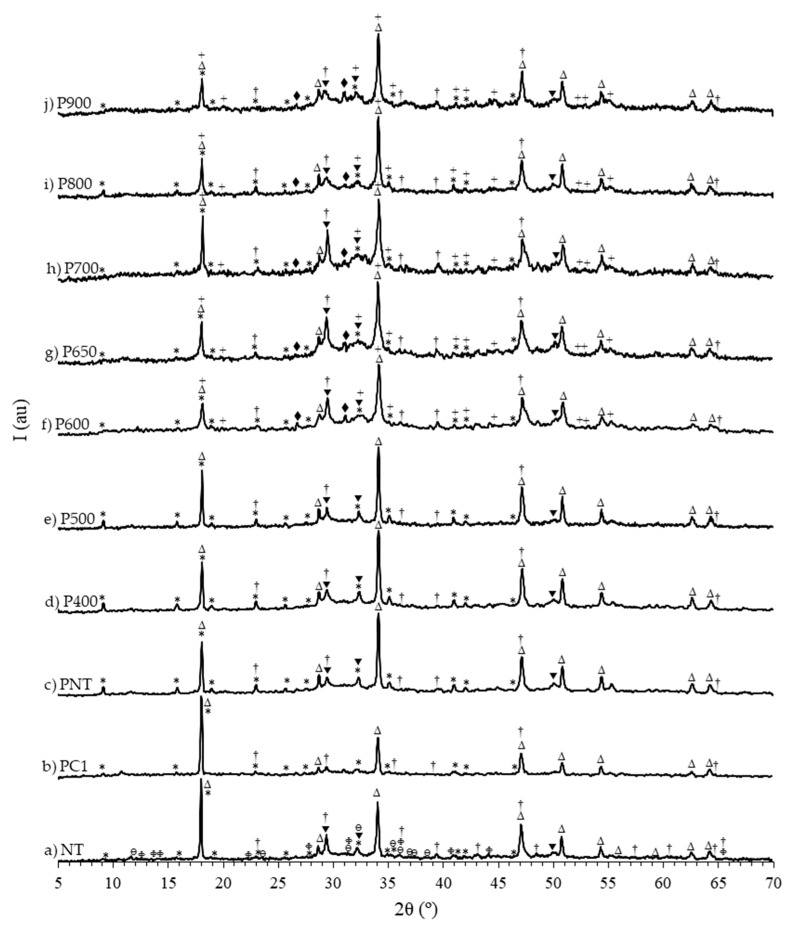
XRD analysis of NT at 90 days and PC1, PNT and RC pastes at 28 days. Δ CH (portlandite); † CaCO_3_ (calcite); * AFt (ettringite); θ C-S-H; ▼ C-S-H; ◆ tobermorite; + C2S; Φ alumohydrocalcite; θ calcium aluminum oxide carbonate hydrate.

Figure 21 presents the XRD results regarding crystalline phases identified in NT and RC pastes at 28 days. Overall, the RC pastes formed hydration products similar to those of NT, confirming the rehydration ability of the thermoactivated recycled cement.

In pastes produced with RC treated above 600 °C, the CH peaks tended to be less intense than those of NT. On the one hand, the CH obtained from rehydration had weaker bonding, as found in the TG analysis (Section 3.3.3). On the other hand, the amount of calcite tended to increase above 500 °C, which resulted in the reduction of CH.

The absence of ettringite peaks in some of the pastes is expected to have resulted from its sensitivity to grinding, as well as to prolonged storage in isopropanol [50]. In general, calcite may have resulted from carbonation occurred during the milling and storage processes. However, given that anhydrous RC treated above 750 °C did not display calcite peaks (Figure 21i–k) and that the pastes were cured in water, the presence of calcite in P800 and P900 indicates that carbonation should have occurred at a different stage. In fact, as pointed out in Section 3.3.3, chemical interactions between hydration products and the solvent may occur, leading to sample carbonation, after hydration arrest with isopropanol, when the cement paste is finely ground [51]. A similar trend was observed between anhydrous PC and PC pastes (Section 3.1.2 and Section 3.3.3).

#### 3.3.5. Nuclear Magnetic Resonance Spectroscopy of Hydrated Recycled Cement Pastes

**Figure 22:**
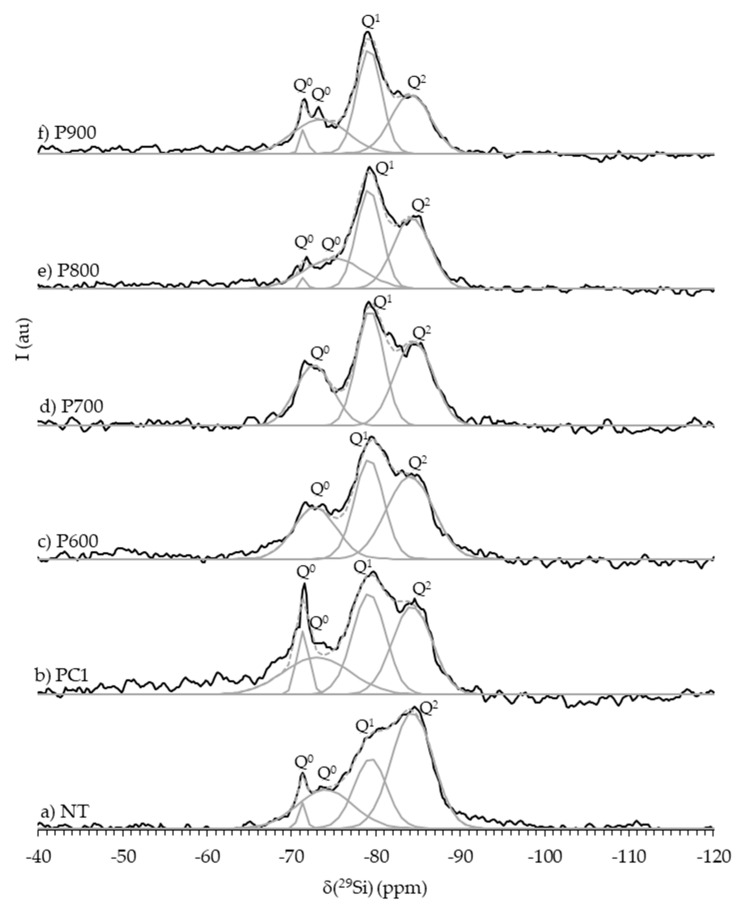
^29^Si-NMR spectra of NT at 90 days, PC1 and RC pastes at 28 days (peak deconvolution is displayed in gray). Q^n^ refers to the number of orthosilicate units (n) attached to a SiO_4_ tetrahedron (Q).

Figure 22 presents the ^29^Si-NMR spectra of NT at 90 days, as well as of PC1 and RC pastes at 28 days. Overall, the deconvolution of RC pastes’ spectra showed Q^0^, Q^1^ and Q^2^ silicate structural units (Table 9), confirming the rehydration of RC.

The Q^0^ peaks correspond to remaining unreacted C_2_S polymorphs, whereas the Q^1^ and Q^2^ peaks represent the progressive development of hydration products, namely C-S-H gel. The hydration degree, *α_H,NMR_* (Table 9), was approximately estimated through Equation (5), assuming that the effect of non-calcium silicates is not significant in the hydration of cement pastes [52]. The mean silicate chain length of C-S-H, *MCL*, was also calculated (Equation (6) [53]).

(5) $$\alpha_{H,NMR}=\;\frac{Q^{1}+Q^{2}}{Q^{0}+Q^{1}+Q^{2}}$$

(6) $$MCL=\;\frac{2Q^{1}+2Q^{2}}{Q^{1}}$$

In Equations (5) and (6), Q^n^ values correspond to the integrated intensities of the corresponding ^29^Si-NMR peaks after deconvolution. These parameters, as well as the Q^2^/Q^1^ ratios are indicated in Table 9. The obtained *MCL* values ranged between 3–6 and are representative of common C-S-H with a C/S ratio higher than 1.2 [54]. The ^29^Si-NMR spectrum of NT presented significantly higher *MCL* (6.12) and Q^2^/Q^1^ ratio (2.06) than PC1 (4.04 and 1.02) and RC pastes (3.75–4.60 and 0.88–1.30). This indicates the formation of a larger amount of C-S-H with longer chain length in NT, which can be explained by the higher maturity and lower w/b (0.55) of this paste.

As discussed in Section 3.1.3, only a small portion of anhydrous Q^0^ phases remained in NT. The spectrum of PC1 also displayed a Q^0^ peak with the same δ(^29^Si) of that found in NT. However, the *MCL* and Q^2^/Q^1^ ratio were significantly lower, due to the higher w/b (0.72) and earlier testing age of PC1. The difference in the estimated *α_H,NMR_* of both pastes confirmed this trend (Table 9). The δ(^29^Si) of the Q^1^ and Q^2^ peaks of the ^29^Si-NMR spectra of RC pastes were similar to those of PC1 and NT and within the range of those commonly reported in cement pastes [10,12,52,55,56,57,58]. Noteworthy is the similarity between the *MCL* and Q^2^/Q^1^ ratios of P700–P800 and those of PC1 (Table 9). This indicates that the type of C-S-H products formed in PC and RC pastes after 28 days was similar. The *α_H,NMR_* was lower in PC1 than in RC pastes (Table 9), which confirms that a higher volume of C-S-H products was formed in the RC pastes. As mentioned, this is related to the higher surface area of the RC, which is also able to develop internal hydration products, contrarily to PC, in which the anhydrous particle core takes longer to be consumed.

The slightly higher *MCL* and Q^2^/Q^1^ ratio of P600 suggests a faster reaction of this material, justified by the higher reactivity of its C_2_S polymorphs. This is in line with the total porosity results (Table 7) and with the trend observed in the mechanical strength tests (Figure 12) of P600 and P650.

Above 800 °C, the *MCL* value and the Q^2^/Q^1^ ratio decreased with treatment temperature, suggesting that the RC particles reacted slower and that lower molecular weight species (shorter chain length) were formed. In addition, the bulk area of the Q^0^ peak in the ^29^Si-NMR spectra of P600 and P700 depicted the lower crystallinity of the calcium silicates, which is in accordance with the ^29^Si-NMR spectra of the corresponding anhydrous RC (RC600 and RC700) presented in Section 3.1.3. Moreover, in conformity with the anhydrous RC (RC800 and RC900), the ^29^Si-NMR spectra of P800 and P900 presented narrower Q^0^ peaks than the other RC pastes, and with δ(^29^Si) similar to those obtained for NT or PC1. Lü et al. [12] also found that the calcined β-C_2_S in RC treated at 900 °C had low reactivity after 28 days and did not divert from its initial state, before hydration. Finally, the *α_H,NMR_* value tended to decrease with increasing treatment temperature above 600 °C, showing that higher hydration of C-S-H was attained in these pastes. As found in previous sections, above 600 °C, RC900 was the least reactive RC within the first 28 days, leading to the lowest *α_H,NMR_*. The *α_H,NMR_* values were slightly higher (~10 %) than those estimated from the TG analysis (which covers all compounds in the paste and not just the calcium silicates). Note that, as mentioned in Section 3.3.3, the *α_TG_* takes into account the CH derived from the decarbonation of compounds, such as lime filler, overestimating the amount of hydration products.

## 4. Conclusions

The present paper presented an extensive experimental research on the influence of the treatment temperature on the rehydration behavior, microstructure and mechanical properties of RC, involving a wide range of treatment temperatures (400–900 °C).

The particle density increased and the particle size was reduced with the treatment temperature, essentially due to C-S-H dehydration and decarbonation process, implying a significant reduction of the specific volume. Above 750 °C, particle binding may contribute to the reduction of RC surface area. The XRD and ^29^Si-NMR analyses of RC confirmed the effective depolymerization of C-S-H and the development of C_2_S polymorphs of varying reactivity, above 600 °C. Up to 500 °C, RC showed slow reactivity due to poor depolymerization during thermal treatment. Between 600–750 °C, the main identified C_2_S polymorph was α’_L_-C_2_S. Above 800 °C, β-C_2_S was also formed and anhydrous phases were more crystalline, and hence, RC reactivity was reduced. The high reactivity of the newly formed compounds above 600 °C was confirmed by the high hydration heat release. Nevertheless, although RC had higher initial hydration heat, its long term reactivity was lower than that of PC.

Despite the high initial reactivity, the setting times of RC increased with the treatment temperature, having been at least twice as high as that of PC. Moreover, the water demand increased with the treatment temperature, having been up to about three times higher than that of PC.

The XRD and ^29^Si-NMR analyses of RC pastes demonstrated the development of similar hydration products to those of PC pastes. Similar *MCL* and Q^2^/Q^1^ ratios were found in RC and PC pastes, suggesting the generation of C-S-H products of the same type.

Contrary to the PC paste, the RC pastes were characterized by two distinct microstructures, one concerning the internal porous RC particles and other related to the surrounded bulk paste. Part of the water is consumed in the development of RC internal products, which implies that the external bulk paste w/b is reduced and the RC grains become closer. Therefore, pastes with RC treated above 600 °C were able to show a more refined porosity than the PC paste of similar total porosity. On the other hand, due to their poor reactivity, pastes with RC treated up to 500 °C had a poor development of external hydration products outside RC particles, leading to pastes of loose microstructure, coarse porosity and low mechanical strength. The pastes with RC treated above 800 °C developed less dense microstructures and C-S-H of shorter chain length, because of their high initial w/b and slower reactivity.

Overall, on based the mechanical and microstructural characterization, optimal treatment temperatures were achieved at around 600–650 °C, which also imply no significant decarbonation during RC production. Compared to PC pastes of equal w/b, the compressive strength of these RC pastes was higher until three days and up to 27% lower at 28 days.

## Figures and Tables

**Table 1:** Properties of the ordinary PC (provided by the supplier (SECIL)).

Parameter		CEM I 42.5 R	Standard
Density (g/cm^3^)		3.07	LNEC E 64 [27]
Blaine specific surface (cm^2^/g)		4437	EN 196-6 [28]
Residue on the 45 μm sieve (%)		6.80	EN 196-6 [28]
Compressive strength of reference mortar (MPa)	1 day	16.8	EN 196-1 [29]
	2 days	28.8	
	7 days	43.6	
	28 days	57.0	
Expansion (mm)		1	EN 196-3 [30]
SiO_2_ + Al_2_O_3_ + Fe_2_O_3_ (%)		19.64 + 5.34 + 3.05	-
CaO + MgO (%)		62.80 + 1.80	-
Free CaO + MgO (%)		0.7 + 0.9	-
Setting time (min)	initial	170	EN 196-3 [30]
	final	280	

**Table 2:** Composition of the produced pastes.

Paste Designation	PC1	PC2	PNT	P400	P450	P500	P600	P650	P700	P750	P800	P900
**Binder**	PC	PC	NT	RC400	RC450	RC500	RC600	RC650	RC700	RC750	RC800	RC900
**w/b**	0.72	0.31	0.43	0.62	0.64	0.68	0.73	0.72	0.72	0.74	0.87	0.91

w/b: water-to-binder ratio; PC: pastes produced with CEM I 42.5R; PNT: paste produced with waste cement without thermal treatment; Pxxx: pastes produced with recycled cement; RCxxx: recycled cement powder; xxx treatment temperature.

**Table 3:** Estimated *L_dh_*, *L_dx_*, *L_dc_*, *W_b_*, α_TG_, CH and CaO contents of PC, NT and RC treated at different temperatures.

Binder Designation	*L_dh_* (wt%)	*L_dx_* (wt%)	*L_dc_* (wt%)	*W_b_* (%)	*α_TG_* (%)	CH (wt%)	CaO (wt%)
PC (CEM I 42.5R)	0.10	0.20	3.07	0.30	1.30	0.82	-
NT	12.96	3.90	3.93	17.37	75.53	18.16	-
RC400	2.80	4.40	4.43	7.20	31.30	18.10	-
RC450	3.00	4.00	4.72	7.00	30.43	16.45	-
RC500	1.20	3.70	5.34	4.90	21.30	15.22	-
RC600	0.90	3.20	4.65	4.10	17.83	13.16	3.67
RC650	1.10	3.20	3.99	4.30	18.68	13.16	4.49
RC700	0.80	3.20	3.75	4.00	17.39	13.16	4.84
RC750	0.80	3.10	3.85	3.90	16.96	12.75	5.02
RC800	0.50	2.00	1.55	2.50	10.87	8.23	11.46
RC900	0.40	2.20	1.51	2.60	11.30	9.05	10.90

*L_dh_*: mass loss on C-S-H dehydration and AFm and AFt decomposition; *L_dx_*: mass loss on CH dehydroxylation; *L_dc_*: mass loss on decarbonation; *W_b_*: non-evaporable water; *α_TG_*: hydration degree; RCxxx: recycled cement powder; *xxx* treatment temperature.

**Table 4:** Isotropic chemical shifts (δ^29^Si) for PC, NT and RC treated at different temperatures.

Paste Designation	Structural Unit	δ(^29^Si) (ppm)	Q^2^/Q^1^
PC	Q^0^	−71.67	-
	Q^0^	−75.40	
NT	Q^0^	−71.27	2.06
	Q^0^	−73.98	
	Q^1^	−79.33	
	Q^2^	−84.31	
RC400	Q^0^	−70.86	0.89
	Q^0^	−74.4	
	Q^1^	−79.33	
	Q^2^	−83.8	
RC600	Q^0^	−71.35	-
	Q^0^	−75.42	
RC700	Q^0^	−71.35	-
	Q^0^	−75.60	
RC800	Q^0^	−71.56	
	Q^0^	−70.60	
RC900	Q^0^	−71.46	-
	Q^0^	−70.81	

*δ(^29^Si)*: isotropic NMR chemical shift of the Si atoms; *Q^2^/Q^1^*: Q^2^-to-Q^1^ integrated intensity ratio.

**Table 5:** Water demand and setting time of PC and RC, and flowability of the pastes.

Paste Designation	Binder	Water Demand (w/b)	Setting Time (min)		Flowability(mm)
			Initial	Final	
PC2	CEM I 42.5R	0.31	85	115	163
PNT	NT	0.43	-	-	154
P400	RC400	0.62	145	265	117
P450	RC450	0.64	185	275	154
P500	RC500	0.68	165	295	182
P600	RC600	0.73	193	275	164
P650	RC650	0.72	215	330	144
P700	RC700	0.72	315	417	149
P750	RC750	0.74	338	375	154
P800	RC800	0.87	360	460	147
P900	RC900	0.91	>720	>1440	145

w/b: water-to-binder ratio; PC: pastes produced with CEM I 42.5R; PNT: paste produced with waste cement without thermal treatment; Pxxx: pastes produced with recycled cement; RCxxx: recycled cement powder; xxx treatment temperature.

**Table 6:** Hardened properties of the studied pastes.

Paste Designation	Binder Designation	w/b	Hardened Density (kg/m^3^)	f_ctm,1d_ (MPa)	CV (%)	f_ctm,3d_ (MPa)	CV (%)	f_ctm,7d_ (MPa)	CV (%)	f_ctm,28d_ (MPa)	CV (%)	f_ctm,90d_ (MPa)	CV (%)	f_cm,1d_ (MPa)	CV (%)	f_cm,3d_ (MPa)	CV (%)	f_cm,7d_ (MPa)	CV (%)	f_cm,28d_ (MPa)	CV (%)	f_cm,90d_ (MPa)	CV (%)
PC1	CEM I 42.5R	0.72	1740	1.8	7	3.7	8	3.8	7	4.5	16	5.6	4	7.8	10	14.0	2	19.0	9	26.5	5	27.4	9
PC2	CEM I 42.5R	0.31	2180	7.0	27	8.2	20	9.2	21	9.7	6	11.1	3	57.7	6	68.6	6	71.7	6	80.1	4	102.8	4
PNT	NT	0.43	1880	-	-	0.2	3	-	-	0.3	6	-	-	-	-	0.3	9	-	-	0.5	10	-	-
P400	RC400	0.62	1640	-	-	0.9	11	-	-	1.0	2	-	-	-	-	2.4	6	-	-	4.08	5	-	-
P450	RC450	0.64	1660	-	-	1.3	9	-	-	1.2	6	-	-	-	-	5.4	5	-	-	6.1	6	-	-
P500	RC500	0.68	1640	-	-	1.2	7	-	-	1.9	5	-	-	-	-	8.0	2	-	-	8.7	5	-	-
P600	RC600	0.73	1730	1.8	18	2.0	9	2.4	6	2.9	9	3.0	2	11.1	6	12.3	2	15.5	5	16.9	5	19.2	3
P650	RC650	0.72	1720	-	-	2.3	7	-	-	2.9	11	-	-	-	-	16.7	3	-	-	19.2	7	-	-
P700	RC700	0.72	1740	1.1	1	2.5	17	2.6	7	3.0	7	3.0	9	4.4	3	13.8	9	15.6	9	18.0	5	21.3	5
P750	RC750	0.74	1720	-	-	2.0	2	-	-	2.9	1	-	-	-	-	10.7	5	-	-	16.5	4	-	-
P800	RC800	0.87	1660	-	-	2.3	13	-	-	2.6	9	-	-	-	-	11.7	6	-	-	18.3	9	-	-
P900	RC900	0.91	1660	-	-	0.5	17	-	-	2.3	7	-	-	-	-	1.4	8	-	-	14.4	6	-	-

w/b: water-to-binder ratio; f_ctm,xd_: paste flexural strength at test age x; CV: coefficient of variation; f_cm,xd_: paste compressive strength at test age x.

**Table 7:** Porosity parameters of PC1, PC2 and RC pastes.

Parameter	PC1	PC2	P450	P500	P600	P650	P700	P750	P800	P900
Total MIP porosity (%)	41.19	12.67	44.40	42.82	39.43	37.49	44.07	42.79	40.65	47.97
Critical pore diameter (μm)	0.083	0.046	0.216	0.216	0.091	0.066	0.091	0.034	0.081	0.059

**Table 8:** Estimated *L_dh_*, *L_dx_*, *L_dc_*, *W_b_*, α_TG_ and CH contents of PC, PNT and RC pastes at 28 days.

Paste Designation	*L_dh_* (wt%)	*L_dx_* (wt%)	*L_dc_* (wt%)	*W_b_* (wt%)	*α_TG_* (wt%)	CH (wt%)
PC1	12.36	4.00	3.86	16.84	73.21	18.43
PC2	11.36	2.60	3.48	14.27	62.04	11.98
PNT	12.66	2.80	3.68	15.46	67.20	11.52
P400	12.46	3.20	4.58	15.90	69.12	14.15
P500	12.56	3.30	4.60	15.86	68.94	13.57
P600	12.13	3.10	5.02	15.60	67.82	14.26
P650	11.93	2.90	5.88	15.79	68.65	15.86
P700	12.36	2.50	5.72	15.84	68.89	14.34
P800	12.46	3.00	4.50	16.74	72.79	17.62
P900	13.36	3.10	3.58	17.39	75.59	16.56

*L_dh_*: mass loss on C-S-H dehydration and AFm and AFt decomposition; *L_dx_*: mass loss on CH dehydroxylation; *L_dc_*: mass loss on decarbonation; *W_b_*: non-evaporable water; *α_TG_*: hydration degree; PC: pastes produced with CEM I 42.5R; PNT: paste produced with waste cement without thermal treatment; Pxxx: pastes produced with recycled cement; RCxxx: recycled cement powder; xxx treatment temperature.

**Table 9:** Isotropic chemical shift *δ(^29^Si)* and calculated value of *α_H,NMR_* and *MCL* of NT, PC1 and RC pastes.

Paste Designation	Structural Unit	δ(^29^Si) (ppm)	Q^2^/Q^1^	*α_H,NMR_*	*MCL*
NT	Q^0^	−71.27	2.06	0.75	6.12
	Q^0^	−73.98			
	Q^1^	−79.34			
	Q^2^	−84.31			
PC1	Q^0^	−71.37	1.02	0.68	4.04
	Q^0^	−73.00			
	Q^1^	−79.16			
	Q^2^	−84.30			
P600	Q^0^	−72.77	1.30	0.76	4.60
	Q^1^	−79.18			
	Q^2^	−84.04			
P700	Q^0^	−72.67	1.02	0.74	4.04
	Q^1^	−79.32			
	Q^2^	−84.51			
P800	Q^0^	−71.53	1.02	0.73	4.05
	Q^0^	−75.00			
	Q^1^	−79.18			
	Q^2^	−84.22			
P900	Q^0^	−71.47	0.88	0.71	3.75
	Q^0^	−73.40			
	Q^1^	−79.20			
	Q^2^	−84.12			

*δ*(*^29^Si)*: isotropic NMR chemical shift of the Si atoms; *Q^2^/Q^1^*: Q^2^-to-Q^1^ integrated intensity ratio.
